# Label-free Quantitative Analysis of Changes in Broiler Liver Proteins under Heat Stress using SWATH-MS Technology

**DOI:** 10.1038/srep15119

**Published:** 2015-10-13

**Authors:** Xiangfang Tang, Qingshi Meng, Jie Gao, Sheng Zhang, Hongfu Zhang, Minhong Zhang

**Affiliations:** 1 State Key Laboratory of Animal Nutrition, Institute of Animal Science, Chinese Academy of Agricultural Sciences, Beijing 100193, China; 2 Institute of Biotechnology, Cornell University, Ithaca, NY 14853-2703, USA

## Abstract

High temperature is one of the key environmental stressors affecting broiler production efficiency and meat yield. Knowledge of broiler self-regulation mechanisms under heat stress is important for the modern scale of poultry breeding. In the present study, the SWATH strategy was employed to investigate the temporal response of the broiler liver to heat stress. A total of 4,271 proteins were identified and used to generate a reference library for SWATH analysis. During this analysis, 2,377 proteins were quantified, with a coefficient of variation ≤25% among technical and biological replicates. A total of 257 proteins showed differential expression between the control and heat stressed groups. Consistent results for 26 and 5 differential proteins were validated respectively by MRM and western blotting quantitative analyses. Bioinformatics analysis suggests that the up- and down-regulation of these proteins appear involved in the following three categories of cellular pathways and metabolisms: 1) inhibit the ERK signaling pathway; 2) affect broiler liver lipid and amino acid metabolism; 3) induce liver cell immune responses to adapt to the high temperatures and reduce mortality. The study reported here provides an insight into broiler self-regulation mechanisms and shed light on the improved broiler adaptability to high-temperature environments.

Most poultry production methods employed around the world involve large numbers of broilers living in controlled environments. Understanding and controlling environmental conditions is crucial for successful poultry production and welfare. High-density cultivation leads to higher ambient temperatures, especially during summer. Genetically improved broilers are more productive than wild *Gallus gallus* but are less adaptable to environmental changes^1^. Exposure to high ambient temperatures and high humidity is known to have a detrimental effect on broiler production efficiency and meat yields^2^. At an ambient temperature of 28 °C, the appetite of broilers decreases by 12% and by as high as 50% when high relative humidity is also present^3^. Therefore, comprehensively understanding the molecular mechanism and metabolic alteration of the physiological responses to heat is critical to improve poultry production efficiency and welfare. Some genetic mechanisms, including the synthesis of molecular chaperones, the generation of reactive oxygen species (ROS), and induction of the antioxidant defense system, have been reported as important indicators of heat stress^4^,^5^.

With the rapid development of gene microarray and high-throughput sequencing technologies, many transcriptomic studies have been conducted using a systems-biology approach to characterize changes in mRNA expression of thousands of genes in different tissues to gain a comprehensive understanding of transcriptomic response to heat stress^6^,^7^,^8^,^9^. Li *et al.* investigated the transcriptome of broiler breast tissue in response to cyclic high ambient temperatures and identified 110 differentially expressed genes involved in the mitogen-associated protein kinase (MAPK), ubiquitin-proteasome, and nuclear factor kappa-light-chain-enhancer of activated B cells (NFKB) pathways^10^. Coble *et al.* used RNA-seq technology for analysis of the transcriptome of the broiler liver under high ambient temperatures and found that high temperatures induced various physiological responses such as decreased internal temperatures, reduced hyperthermia, and cellular reactions promoting apoptosis, tissue repair, and regulating perturbed cellular calcium levels^1^. These studies show that animal adaptations to heat stress apparently depend on activation of the hypothalamic-pituitary-adrenal axis and the orthosympathetic nervous system as well as the expression of numerous stress-related genes. Since mRNA molecules only carry genetic information on transcriptomic expression, they may not directly reflect the abundance of proteins and yield no post-translational modification information for any given proteins, which are more directly involved in cellular function and metabolism. Hence, the research on molecular mechanisms for heat stress at the mRNA level alone is not sufficient because there are many different splicing and post-modifications following mRNA translation that would affect the final functions of genes or proteins^11^,^12^,^13^. Therefore, it is necessary to analyze protein changes under heat stress.

The rapid development of proteomics technologies in combination with the vast amount of available *Gallus gallus* genome sequence information provides an unprecedented opportunity for proteomics profiling in chickens. Proteomic analysis has become one of the popular strategies for identifying proteins and pathways that are crucial to stress response^4^. The quantitation techniques applied in proteomics are usually classified as direct LC-MS/MS acquisition (label-free quantitation) based on extracted precursor signal intensities of peptides or on spectral counting which simply counts the number of spectra identified for a given peptide in different biological samples, or by the use of stable isotope labeling prior to LC-MS/MS acquisition^14^,^15^. Relative quantitation methods, such as ICAT, SILAC, TMT or iTRAQ, use stable isotope-based labeling to quantify proteins and compare the results as relative peptide abundances in different samples using either precursor ions in survey MS spectra or specific reporter ions in MS/MS spectra^15^,^16^,^17^,^18^,^19^. In selected/multiple reaction monitoring (SRM/MRM), targeted proteins may be relatively quantitated based on selected ion pairs for each of the target peptides. Meanwhile stable isotope labeled reference peptides may be used allowing for an absolute quantitation^15^,^20^,^21^. SRM/MRM is carried out by acquiring predefined pairs of precursor and product ion masses, referred to as transitions, several of which constitute a definitive assay for the detection of a peptide in a complex sample^22^. Shotgun proteomics and targeted proteomics exhibit different and largely complementary studies and analysis performance, which have been extensively discussed^22^,^23^. Specifically, shotgun proteomics is the optimal method for discovering the maximum number of proteins, although it will often sacrifice quantitation accuracy and throughput for complex samples^22^,^24^. In contrast, targeted proteomics is well suited for reproducibly and accurately quantifying sets of known, specific proteins in many samples but is limited to measurements of a few thousand transitions per LC-MS/MS run^25^. SWATH (Sequential window acquisition of all theoretical spectra)-MS is a new label-free, quantitative proteomics analysis strategy that combines the advantages of both shotgun and targeted proteomics. This new strategy is able to quantify thousands of proteins in a single measurement; the data are acquired on a fast, high-resolution Q-TOF instrument by repeatedly cycling through sequential isolation windows over the whole chromatographic elution range^22^,^26^,^27^.

The liver, one of the most vital organs in the body, plays a critical role in metabolism, digestion and immune defense. In energy metabolism, the liver exhibits a wide range of functions, such as glycogenolysis and glycogen synthesis, protein metabolism, hormone production, and detoxification^4^. The liver is also more susceptible to oxidative stress than the heart during acute heat exposure in broiler chickens^5^. In the present study, we employed the SWATH-MS workflow to conduct proteomic profiling of the broiler liver in response to heat stress. Following statistics and bioinformatics analyses of the identified candidate proteins responsible for heat stress, several important candidate proteins were further validated by Western blotting and empirically confirmed by MRM-based peptide quantitative and metabolite quantitative analyses. Our results provide insight into the complex molecular mechanisms associated with heat stress response in the broiler liver and further shed light on potential heat stress mechanisms.

## Results

### Experimental design and workflow

**Figure 1:**
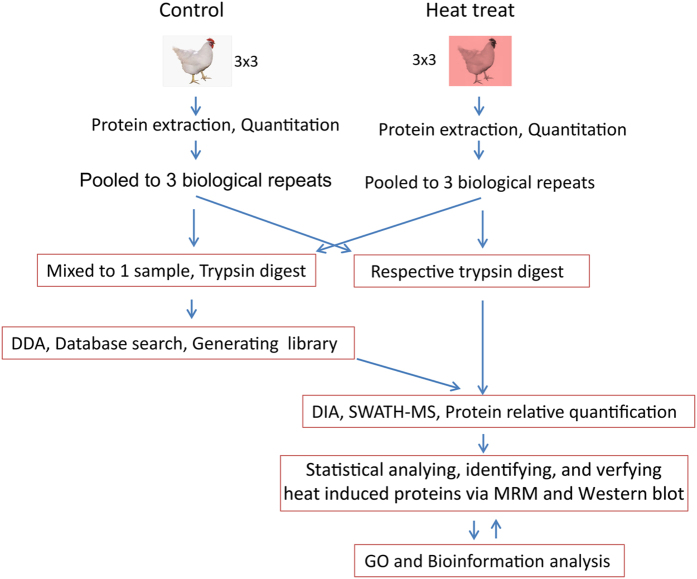
Experimental design and workflow for the broiler liver quantitative proteomics analysis using the SWATH strategy. First, 21-day broilers were randomly divided into two groups, each containing nine broilers. After being subjected to heat treatment for 72 hours, the broiler livers were collected, and proteins were extracted and quantitated in the control group of broilers. Three liver protein samples were pooled in equal amounts as one biological replicate. A total of three biological replicates and three technical replicates were designed and conducted in the study. One sample mixed with the above six samples was used to generate a reference library. The six individual samples were analyzed with SWATH 2.0. All of the data were used for statistical analysis and confidently quantified proteins across all 6 samples in both groups were obtained for the subsequent GO, bioinformatics analysis, and validation experiments by MRM and Western blotting.

The main objective of this study was to identify heat response proteins during heat stress treatments to gain a better understanding of the underlying metabolic processes and molecular mechanisms involved. SWATH 2.0 label-free proteomics quantitative technology was utilized to obtain a global view of the proteome dynamics and changes associated with the heat response of broiler livers. The experimental design and workflow are illustrated in Fig. 1. The broilers (Arbor Acres) were divided into two groups containing nine broilers each. The experiment included a total of three biological replicates and three technical replicates for each of the biological samples. Each biological sample was composed of three individual broiler liver samples which were equally mixed. Following the large-scale identification and functional categorization of differentially expressed proteins, Western blotting and MRM validation analysis were conducted for the control and heat treatment groups.

### Generating a high-quality reference spectral library for SWATH quantitative analysis

Protein quantitation via SWATH was performed using the reference spectral library based on information extracted from the information dependent acquisition (IDA) files. The reference spectral library encompassed all peptides and transitions of the identified proteins. To maximize the number of proteins for SWATH quantitation, we cascaded two analytical columns to increase the separation efficiency, which would decrease the number of co-eluting peptides. We also analyzed the mixed sample using the TripleTOF® 6600 system for IDA analysis with variable range MS scans in three runs: *m*/*z* 350–1,250 (Run 1), 350–750 (Run 2) and 745–1,250 (Run 3). We collected 123,812, 57,096 and 85,572 high-quality MS/MS spectra, respectively (Supplemental Figure 1 A–C). After searching the *Gallus gallus* database using ProteinPilot 5.0 at a 1% critical false discovery rate (FDR), we identified 3,904 proteins and 30,293 peptides in Run 1. Due to the high number of co-eluting peptides, only the top 40 parent ions per cycle were acquired via MS/MS, suggesting low-abundance peptides may not be selected for MS/MS. Therefore, we used the low and high *m*/*z* ranges to analyze the same mixed samples separately. Then, we combined all spectra obtained from the three runs prior to the database search. The number of identified proteins was increased to 4,271 (Supplemental Table 1), respectively, at a 1% FDR. Approximately 50% of the proteins were identified based on more than five peptides (Supplemental Figure 1D).

### Quantification and statistical analysis of heat-stress-induced proteins

In order to analyze broiler liver protein changes under heat stress, we applied the SWATH strategy to quantify all proteins in control and heat group samples.

**Figure 2:**
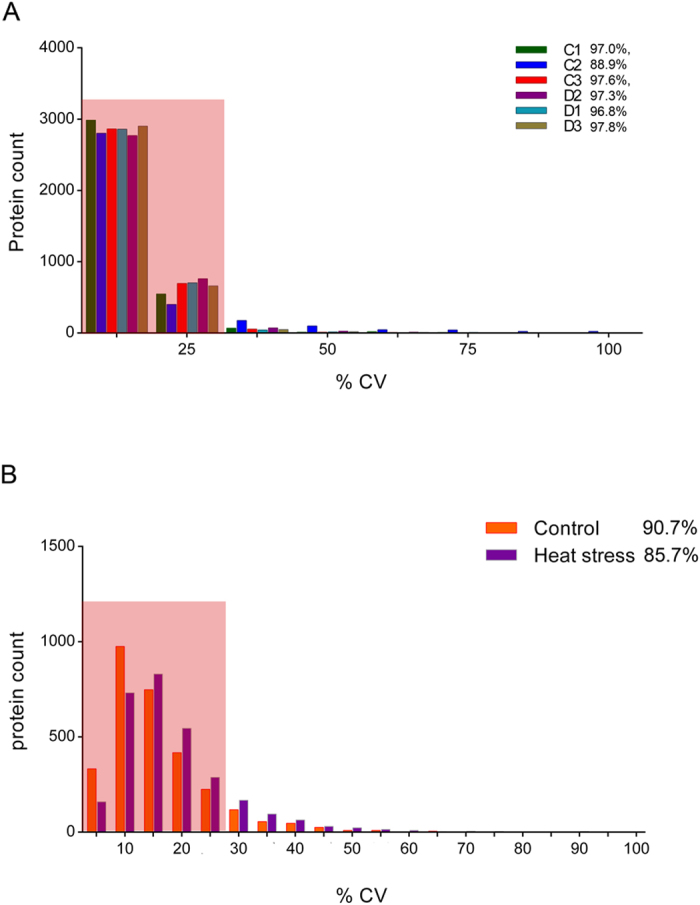
Statistical analysis of the quantitative reproducibility of three biological and technical replicates of the control and heat treatment samples. (**A**) Histogram plots for distribution of the coefficients of variation (CVs) in biological replicates and technical replicates. More than 88.9% of the proteins have quantitative CVs under 25% among the technical replicates. The shadow in A indicates the proteins (CVs ≤ 25%) that were used for analyzing the reproducibility among biological replicates. Similarly, (**B**) shows the distribution of coefficients of variation (CVs) among biological replicates. Greater than 85.7% of the proteins have quantitative CVs under 25% among the biological replicates. The shadow in B indicates the proteins (CVs ≤ 25%) that were used to further analyze the differential proteins induced by heat stress.

Following ion extraction, peak alignment and normalization were performed using Peakview 2.0 software and the above reference spectral library, resulting in quantitative information for 3,646 proteins (Supplemental Table 2) in all 17 runs (data from technical replicate of the C2 biological sample was lost due to instrument malfunction). After statistical analysis, a summary of the protein identification results is presented in Table 1. Among the three technical replicates, the percentages of proteins whose quantitation showed a coefficient of variation (CV) ≤25% in the quantitative data were 97.0%, 88.9%, 97.6%, 97.3%, 96.8% and 97.8% (Fig. 2A). These results show that the SWATH strategy used in this study delivers high throughput and reproducibility for protein quantitation. To stringently analyze the quantitation data, we selected 2,979 proteins that had been quantitated in all 3 biological replicate samples for further analyses (Supplemental Table 3). We then compared reproducibility among the biological replicates in each group and found that the percentage of quantitated proteins (CV ≤ 25%) was 90.7% in the control group and 85.7% in the heat treatment group (Fig. 2B). Finally, 2,377 proteins were obtained with low coefficients of variation (CV ≤ 25%) in comparison of the protein changes between the control and heat treatment groups (Supplemental Table 4). To further confirm the statistical significance of the three biological replicates data, we applied an analysis of variance to determine quantitative reproducibility. As shown in Supplemental Figure 2, the minimum R^2^ value for two biological repeats was 0.9816 in the control group and 0.9810 in the heat treatment group, suggesting the quantitative SWATH data from replicates were highly reproducible.

**Figure 3:**
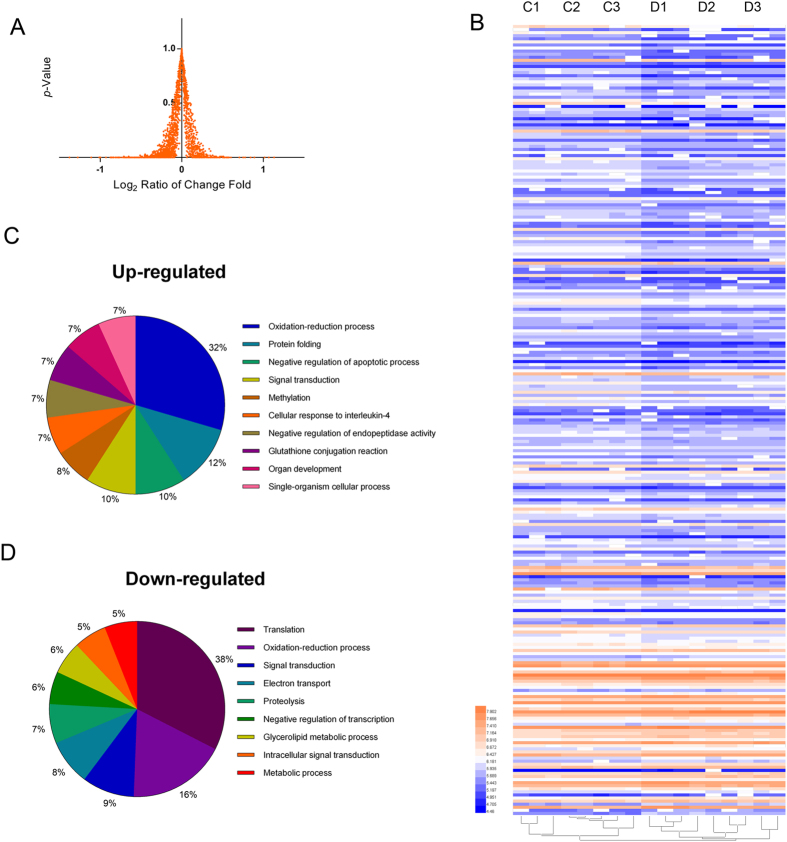
Statistical analyses of the biological functions for 257 differentially expressed proteins induced by heat stress. (**A**) The distribution of p-values and fold changes (log_2_) in 2,377 quantitative proteins between the control and heat treatment groups. A total of 257 proteins were selected as different proteins induced by heat stress, which exhibited a *p*-value < 0.01 and a fold change >1.3. (**B**) Heat map analysis of 257 proteins among three biological replicates between the control and heat treatment groups. The log_10_ value of the MS signal intensity is shown. (**C**,**D**) show the proportions of biological functions among 55 up-regulated proteins and 202 down-regulated proteins. These up-regulated proteins were mainly involved in the oxidation-reduction process, protein folding, signal transduction and the negative regulation of apoptotic process. The down-regulated proteins were involved in protein translation, the oxidation-reduction process and signal transduction.

To identify differentially expressed proteins, a *t*-test analysis was applied, and fold-changes and *p*-values were used to rank and filter the quantitative data (Fig. 3A and Supplemental Table 4). The fold change cutoff at >1.3 was selected based on the standard deviation (Log_2_ = 0.4) from the normal distribution fit at 95% confidence using the Easy-Fit program (MathWave Technologies, http://www.mathwave.com) for the 2377 quantified proteins (Supplemental Table 4). Differentially expressed proteins were defined as those that showed a fold change of greater than 1.3 in relative abundance and a *p*-value < 0.01. In total, 257 proteins differentially expressed between the control and heat treatment groups were identified in the broiler livers, which included 202 down-regulated proteins and 55 up-regulated proteins (Supplemental Table 5). The intensity changes of the differentially expressed proteins are shown as a heat map in Fig. 3B. All of the differentially expressed proteins were functionally categorized using Blast2GO®^28^, a web-based bioinformatics tool that groups proteins based on their GO annotations (Supplemental Table 5). In the present study, proteins were classified according to biological processes at a GO annotation level of 3, and 10 functional categories were selected to cover the datasets. Figure 3C,D show the GO predictions for up- and down-regulated proteins respectively. The majority of up-regulated proteins identified in this study fell under the categories of oxidation-reduction processes, protein folding, signal transduction and negative regulation of apoptotic processes. The majority of the down-regulated proteins identified were assigned to the categories of protein translation, oxidation-reduction process and signal transduction.

### Confirmation of heat-stress-induced candidate proteins via MRM and Western blotting analysis

MRM is considered to be one of the best quantitation methods for targeted proteins based on mass spectrometry with high accuracy, reproducibility, selectivity and dynamic range. Thus, MRM is typically used to verify and validate differentially expressed proteins during or after the proteome discovery stage^29^,^30^. To validate the identification of heat-responsive proteins from SWATH-MS profiling analysis, we used both MRM and classical Western blotting analyses to assess the expression of proteins whose abundance changed in response to heat stress, as determined by SWATH.

**Figure 4:**
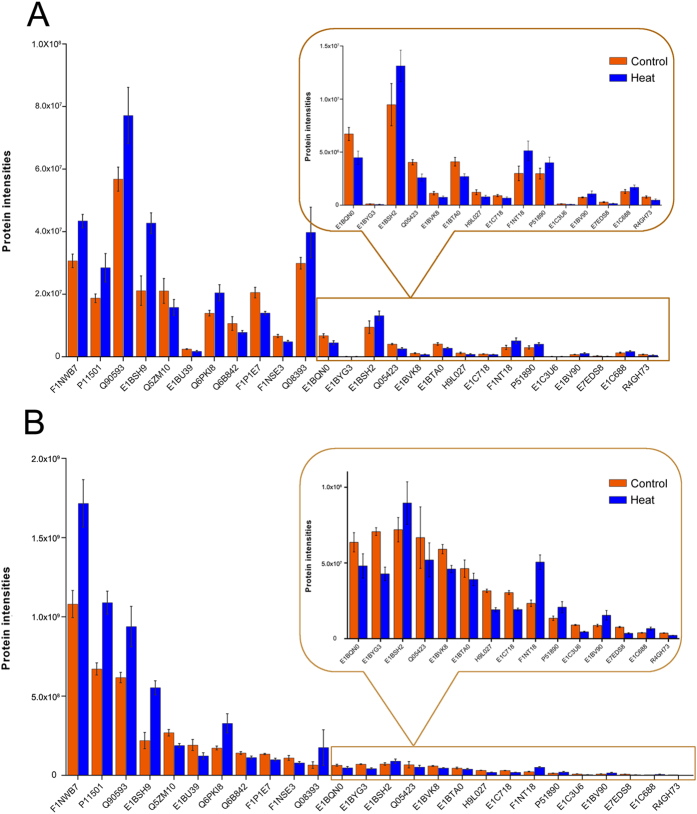
Comparison and verification of the quantitative results for different proteins from the SWATH and MRM analyses. (**A**,**B**) Comparison of the signal intensities of twenty-six different proteins obtained from the SWATH and MRM quantitative analyses, respectively. All of the analyses showed similar quantitative results for twenty six proteins and further verified the accuracy of the SWATH quantitative results. The histogram in rectangle was zoomed in to show the detail of low intensity proteins.

**Figure 5:**
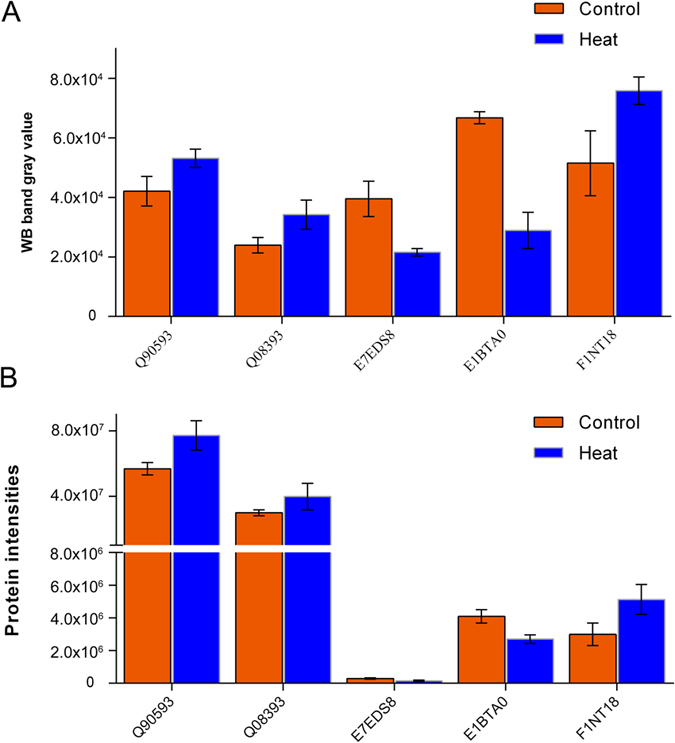
Comparison and verification of the quantitative results for different proteins from the SWATH, MRM and Western blotting analyses. (**A**,**B**) Comparison of the signal intensities of five different proteins obtained from the Western blot and SWATH analyses, respectively. All of the analyses showed similar quantitative results for these five proteins, which confirmed the accuracy of the SWATH quantitative results.

Twenty-six proteins (Supplemental Table 6) were selected for MRM analysis, which are involved in oxidation-reduction, ERK2 interactors, fatty acid metabolism, amino acid metabolism and cell inflammatory responses. We applied t-test to analyze and compare MRM data of Control and Heat treatment groups. As shown in Fig. 4A,B, the SWATH and MRM analyses revealed similar trends for all of these proteins in the control versus the heat treatment group. To further confirm the protein changes, we selected five proteins and applied the classical method for relative protein quantification by Western blotting to validate their abundance in the control and heat treatment groups. The gray values of each protein band were calculated with Quantity One Software and illustrated as a histogram (Fig. 5A). As shown in Fig. 5A,B, the relative abundance of the five proteins was similar to the results from the SWATH analyses (Fig. 5B). All above data indicated that the results from both validation experiments were consistent with the initial discovery result from SWATH analysis, supporting the notion that the SWATH quantitative strategy is a high-throughput, accurate and efficient approach for large-scale quantitative proteomics.

## Discussion

One of the challenges of global quantitative proteomics analyses using existing high-throughput shotgun technologies is the potential for poor data reproducibility and reliability due to biological and technical variations. Therefore, we performed a careful statistical assessment with stringent data filters to ensure excellent reproducibility and reliability were achieved. In the reference library for the SWATH analysis, a total of 4,271 proteins and 36,073 peptides were confidently identified from the mixed protein samples, and three different mass ranges of MS scans in IDA analysis were run without sample prefractionation. A total of 3,646 proteins were quantified across all three biological replicates in both groups using the SWATH strategy (Supplemental Table 2). Among these proteins, more than 88.9% of the CVs were under 25% among the technical replicates, and 85.7% of the CVs were under 25% among the biological replicates (Table 1). The R^2^ values for the biological triplicates in the variance analysis were above 0.98 (Supplemental Figure 2). These analyses showed that the quantitative proteomics data are highly reproducible and reliable, while the subsequent MRM and Western blotting validation analyses confirmed the reliability of these quantitative results. Therefore, a ≤25% CV filter was applied to get a final, high confidence, quantitative proteome for subsequent bioinformatics analysis. Though the fold changes and the relative intensity varied to some degree between the SWATH and MRM analyses (Fig. 4A,B), the change tendencies of the proteins were consistent. For example, the intensity of P11501 was higher than that of Q90593 in the SWATH analysis but lower than that of Q90593 in the MRM analysis. The minor discrepancy between the two is probably because different numbers of quantitative peptides among these proteins were used for the MRM and SWATH analyses.

Considering the vast individual differences among the 18 broilers, we randomly pooled three samples as one biological replicate for each group to minimize this variation. In the subsequent protein change analysis, approximately 89.1% of the proteins did not show significant changes (*p*-value > 0.01 and fold change <1.3), and the maximum fold change was 2.63. These results indicated that the pooled sample did reduce individual differences and enhanced identification of candidate proteins with observed changes in protein abundance responsible for heat stress. Therefore, we used a *p*-value < 0.01 and fold change >1.3 as the threshold parameters to select proteins for the subsequent functional analysis.

To explain the biological events related to heat stress at the molecular level, we performed a functional enrichment analysis of the proteins that were differentially expressed in the liver using the Database for Annotation (Blast2GO 3.0)^28^. As shown in Fig. 3C, the functions of oxidation-reduction and protein folding were mainly associated with up-regulated proteins. In many previous reports, heat shock proteins (HSPs) were induced by heat and other stressors involved in the folding and unfolding of other proteins^31^. In our quantitative SWATH analysis, we successfully obtained quantitative information for HSP70 and HSP90 which were expressed at higher levels in response to heat stress alone. Additionally, the oxidation-reduction balance in liver cells was always shifted by heat stress, resulting in a high ROS level and GSH/GSSG ratio^32^. A number of oxidation-reduction-related proteins/enzymes were found among both up-regulated and down-regulated proteins (Fig. 3C,D). Under heat stress, the body usually shuts down normal protein synthesis to some degree, shifting to synthesizing heat shock proteins or transcription factors and activating the inflammatory response^33^,^34^. A series of proteins involved in protein translation were down-regulated. This observation provides further proof that the differentially expressed proteins revealed functional and metabolic changes in the broiler liver.

**Figure 6:**
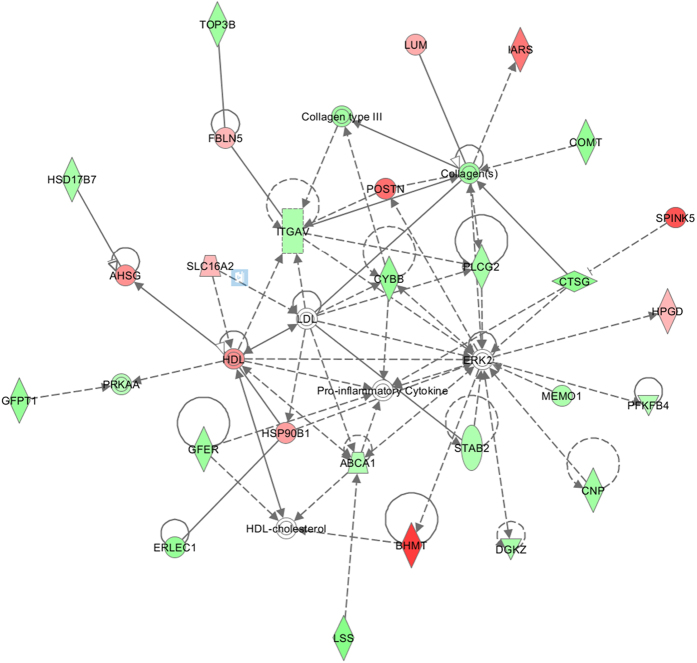
IPA was used to determine the pathways of the different proteins. The results show that heat stress inhibited the ERK2 signaling pathway to regulate cell survival.

**Figure 7:**
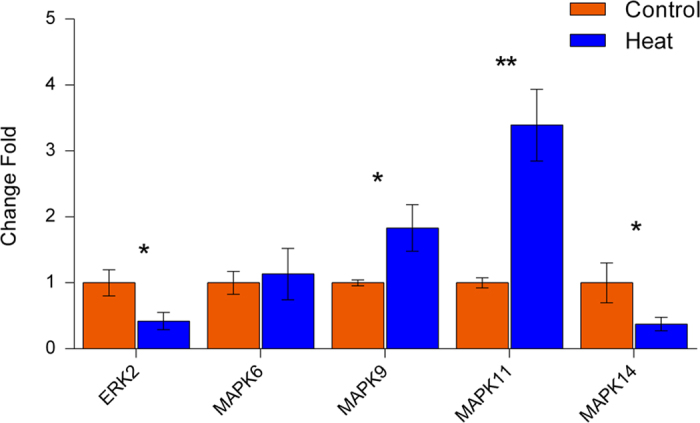
Transcriptional analyses of five *MAPK* genes using RT-PCR. The result showed that these gene expressions were significantly affected except *MAPK6*. The expressions of *ERK2* and *MAPK14* were down-regulated, while the expressions of *MAPK9* and *MAPK11* were up-regulated under heat stress. (*p < 0.05, **p < 0.01).

Heat stress is widely considered to be a major extracellular stimulus which induces protein denaturation and interrupts critical cellular processes, thus resulting in apoptosis and cell death^35^. Extracellular signal-regulated protein kinases (ERK) are members of the mitogen-activated protein kinase (MAPK) superfamily that can mediate cell proliferation and apoptosis^36^. In our SWATH analysis, fourteen proteins (Table 2 and Fig. 6) were identified which are capable of interacting with ERK2. Previous reports showed that some down-regulated proteins, such as ABCA1, MEMO1 and PLCG2, could inhibit ERK1/2. In mouse macrophages, the homozygous presence of a mutant mouse *ABCA1* gene (knockout) was shown to increase the phosphorylation of mouse ERK1/2 protein in mouse macrophages^37^. Interference with human IGF1R mRNA by siRNA decreases the activation of human ERK1/2 in MCF7 cells, which is mediated by the MEMO1 protein^38^. The heterozygous presence of a mutant *PLCG2* gene (knockout) in mice decreases the rate of activation of mouse ERK1/2 in B lymphocytes^39^. Additionally, inhibition of ERK1/2 may increase/decrease downstream protein expression. For example, inhibition of active human ERK in Hct 15 cells by PD98059 increases expression of the human HPGD protein^40^ while inhibition of mouse ERK1/2 using U0126 decreases the expression of mouse CYBB^41^. Similar to previous reports, the homologous proteins of HPGD and CYBB (R9PXN7 and A7E3K8) identified in this work, were found to be up-regulated and down-regulated, respectively. These analyses indicate that heat stress appears to induce inhibition of ERK2 (MAPK1). We analyzed five genes of the MAPK superfamily *(MAPK1, MAPK6, MAPK9, MAPK11 and MAPK14*) at the transcription level using RT-PCR (Fig. 7). The results showed the gene expressions were significantly affected except *MAPK6*. The expressions of *ERK2* and *MAPK14* were down-regulated, while the expressions of *MAPK9* and *MAPK11* were up-regulated under heat stress. These results support our prediction that heat stress could induce inhibition of ERK2 signal pathway, while ERK2 was a key regulatory factor in apoptosis and regulating cell survival. Moreover, the changes of expression of *MAPKs* suggested that heat stress may affect the protein phosphorylation in the apoptosis process.

Sphingolipids are known to play a crucial role in cell response to heat stress^42^,^43^. Heat stress may induce rapid, transient increases in sphingolipid synthesis, leading to increases of 2 to 100 fold in yeast cells^42^. In addition, the hydrolysis of complex sphingolipids may also participate in stress-initiated ceramide formation^44^. In this analysis, we identified three proteins (P2RX1, SMPD4 and KIT) involved in ceramide synthesis (Table 2). P2RX1 takes part in the signaling pathways involved in ATP-mediated apoptosis, and high levels of P2RX1 can stimulate late *de novo* ceramide synthesis and mitochondrial alterations in thymocytes^45^. Phosphodiesterases (SMPDs) catalyze the hydrolysis of membrane sphingomyelin to form ceramide^46^, and down-regulation of KIT can increase the production of ceramide^47^. Ceramide has been suggested to play important roles in cell cycle arrest, apoptosis, inflammation, and the eukaryotic stress response^46^.

Normally, the fatty acid synthesis pathway is responsible for *de novo* lipogenesis, which stores excess energy as fatty acids in adipose tissues in a NADPH-dependent manner, utilizing acetyl-CoA and malonyl-CoAs as the base molecules^48^. The regulation of *de novo* lipogenesis is influenced by the health status of the animal. Decreases in acetyl-CoA-carboxylase in adipose and liver tissues have been noted in heat-stressed pigs^49^. Acetyl-CoA-carboxylase catalyzes the first step of the synthesis of fatty acids, which is a key point of potential change in lipid metabolism. In this analysis, we found that two acyl-CoA thioesterases (ACOT1 and ACOT8) exhibited decreased expression in the heat treatment samples. We also identified 19 up-/down-regulated proteins (Table 2) involved in fatty acid synthesis, elongation and oxidation, which indicates that in the broilers, fatty acid synthase was decreased to save energy to maintain the basic cell survival needs.

**Figure 8:**
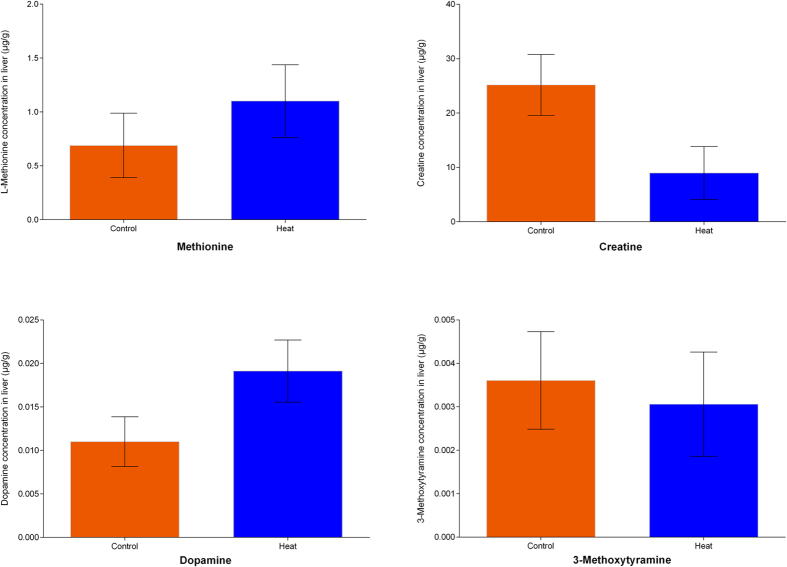
Quantitated analyses of metabolites involving amino acid metabolism. The amount of methionine, creatine, dopamine and 3-methoxytyramine in liver tissue were quantified using LC-MS/MS. Results showed that the abundance of methionine and dopamine were increased, while creatine and 3-methoxytyramine were decreased. The tendency of the above metabolites was consistent with the predicted protein changes.

Oxidative stress induced by high temperatures is responsible for damage to macromolecules, such as lipids, proteins, carbohydrates, and DNA, through the generation of ROS^50^. Methionine, as an antioxidant or pro-oxidant, could reduce ROS damage, and high expression of betaine-homocysteine methyltransferase (BHMT) under heat stress could increase methionine synthesis from betaine to reduce broiler mortality. We also found that guanidinoacetate N-methyltransferase (GAMT) was down-regulated (−1.316 fold), leading to decreased transformation of methionine to creatine (Table 2). The neuro-related substance catecholamine, derived from the amino acids tyrosine and phenylalanine, can stimulate sympathetic nerves and increase the heart rate, blood pressure, and blood glucose levels to help decrease the body temperature of broilers. Dopa decarboxylase (DDC) and phenylalanine hydroxylase (PAH) catalyze catecholamine synthesis from phenylalanine and tyrosine, while catechol-O-methyltransferases (COMT) can degrade catecholamine to 3-methoxytyramine^51^. In our analysis, we found that both DDC (+1.458 fold) and PAH (+1.467 fold) were up-regulated, while COMT was down-regulated, indicating that these changes could promote catecholamine accumulation. In order to observe the change of metabolites involving amino acid metabolism processes induced under heat stress, we conducted quantitation of methionine, creatine, dopamine and 3-methoxytyramine in liver tissue using LC-MS/MS. As shown in Fig. 8, both methionine and dopamine were increased, while creatine and 3-methoxytyramine were decreased. The change of the 4 metabolites was well consistent with the observed change of the three relevant enzymes in response to heat stress by SWATH analysis, proving an additional validation of our SWATH data at the downstream metabolite level.

In poultry, several studies have investigated the effects of heat stress on the immune response in recent years, and their results showed that there were fewer intraepithelial lymphocytes and IgA-secreting cells in the intestinal tract of laying hens under heat stress^52^. In addition, they revealed that the antibody response and the phagocytic ability of macrophages were reduced in abdominal exudate cells in broilers under heat stress^53^. Interestingly, we identified two proteins (HSP90AA1 and LUM) that were up-regulated in the heat treatment samples and that could positively regulate the phagocytic ability of macrophages. We also found three proteins (PRKAA1, LYN and ABCA1) down-regulated in heat stressed samples which may negatively regulate the phagocytic ability of macrophages^54^,^55^,^56^. Phagocytosis is the process by which microbial pathogens and necrotic cells are engulfed by macrophages and neutrophils. The importance of phagocytosis for necrotic cell clearance is of particular relevance to systemic inflammatory diseases which are associated with environmental stress. It has been reported that hypoxia causes an increase in phagocytosis by macrophages in a HIF-1α-dependent manner^57^. Uptake of apoptotic cells by macrophages can also promote cell turnover through transforming growth factor beta1 (TGFB1)^58^,^59^. Additionally, under heat stress, the process of phagocytosis in broilers plays a fundamental role in determining the subsequent adaptive immune response towards the phagocytosed materials as well as in orchestrating the process of regeneration. Furthermore, phagocytosis of dying cells, especially early apoptotic cells in an anti-inflammatory context, plays a vital role in maintaining immunological tolerance against cell-associated antigens^59^.

## Conclusion

Our in-depth quantitative proteomic data substantially expand the proteome coverage of broiler liver tissues and provide new insights into broiler self-regulation mechanisms under heat stress. We identified 257 differentially expressed proteins in liver tissues after heat treatments using the high-throughput, label-free quantitative strategy of SWATH and the results were validated and confirmed by MRM, Western blotting and quantitative metabolite analyses. These proteins were mainly involved in protein translation, the oxidation-reduction process, protein folding, signal transduction and the negative regulation of apoptotic processes. The up-/down-regulation of these proteins may inhibit the ERK signaling pathways, affect lipid and amino acid metabolism in the broiler liver, and induce liver cell inflammatory and immune responses to adapt to high-temperature environments and reduce mortality. Our quantitative proteomics data constitute a proof-of-concept of molecular details during heat stress, where the programmed activation of the proteome matches the observed physiological changes. The identification of biological regulation and key node proteins will allow for further functional analysis of genetic manipulation to be performed. Our results contribute to a broader understanding of broiler self-regulation mechanisms and the use of feed additives to improve broiler adaptability to high-temperature environments.

## Methods

### Experimental animals and long-term heat stress treatment

This study was approved by Animal Welfare Committee of Institutes of Animal Sciences, Chinese Academy of Agricultural Sciences (IASCAAS). All experiments were carried out in strict accordance with the recommendations in the Guide for the Care and Use of Animals of the Chinese Academy of Agricultural Sciences and approved by the Animal Welfare Committee of IASCAAS.

Broiler chicks (Arbor Acres) (*Gallus gallus domesticus*) were purchased from a local hatchery at one day of age and kept in an environmentally controlled room. The brooding temperature was maintained at 35 °C for the first two days and then decreased gradually to 26 ± 1 °C with 60% relative humidity until the 21^st^ day. A total of 18 weight-matched broilers were selected and randomly divided into two groups. The control group was kept at 26 ± 1 °C with 60% relative humidity. The heat treatment group was maintained at 32 ± 1 °C with 60% relative humidity. Following treatment for 72 hours, all of the broiler livers were collected and stored at −80 °C.

### Protein extraction and digestion

All of the chemicals used for protein extraction and digestion were of analytical grade, and Milli-Q water was employed in all buffers and solutions. Protein extraction and digestion were performed by primarily following the Filter Aided Sample Preparation (FASP) protocol^60^. Approximately 0.1 g of liver tissue was homogenized in 1 ml of an extraction buffer (50 mM Tris-HCl, 4% SDS, 100 mM DTT, pH 7.6) for 5 min using a homogenizer (Pro 200, Pro Scientific, USA). The lysates were vortexed at room temperature using the maximum speed for 5 min and were then boiled at 95 °C for 5 min. Then, the lysates were centrifuged at 20,000 × *g* at 25 °C. Protein quantification was performed using the Bradford method. Three different protein samples from three individuals in the same group (Control or Heat treatment) were pooled together in equal amounts as one biological replicate for proteomic analysis. For all of the samples, three biological and three technical replicates were performed. The protein samples were reduced and alkylated with DTT and iodoacetamide (IAA) and digested with trypsin using the FASP method^61^. All of the peptide samples were collected for mass spectrometry analysis.

### Generation of the reference spectral library

Approximately 4 μg of protein from each of 18 samples was pooled together as a mixed sample for tryptic digestion and subsequent IDA analysis. The resulting list of protein/peptides was used for construction of the SWATH reference spectral library. The sample was analyzed via reverse-phase high-pressure liquid chromatography electrospray ionization tandem mass spectrometry (RP-HPLC-ESI-MS/MS) using a TripleTOF® 6600 mass spectrometer (AB SCIEX; Framingham, US). The mass spectrometer was coupled to a nanoLC Eksigent 425 system (AB SCIEX; Framingham, US). RP-HPLC was performed with a trap and elution configuration using a Nano cHiPLC Trap column (200 μm × 0.5 mm ChromXP C18-CL 3 μm 120 Å) and two cascade nano columns (75 μm × 15 cm ChromXP C18-CL 3 μm 120 Å). The reverse-phase LC solvents included solvent A (95% water + 5% DMSO + 0.1% formic acid) and solvent B (95% acetonitrile + 5% DMSO + 0.1% formic acid). The sample was loaded in the trap column at a flow rate of 2 μl/min for 10 min using 100% solvent A and eluted at a flow rate of 300 μl/min using a stepwise gradient (0–1 min, 95% A; 1–90 min, 76% A; 90–120 min, 50% A; 120–135, 50% A; 135–135.5 min, 20% A; 135.5–150 min, 20% A; 150–155.5 min, 95% A; 155.5–165 min, 95% A).

The samples used to generate the SWATH-MS spectral library were subjected to three runs of information-dependent acquisition (IDA). For these experiments, the mass range for MS scan was set to *m*/*z* 350–750, 745–1,250 and 350–1,250, and the MS/MS scan mass range was uniformly set to *m*/*z* 100–1,500. Using the mass spectrometer, a 0.25 s survey scan (MS) was performed, and the top 40 ions were selected for subsequent MS/MS experiments employing an accumulation time of 0.05 s per MS/MS experiment for a total cycle time of 2.25 s. The selection criteria for parent ions included an intensity of greater than 150 cps and a charge state ranging from +2 to +4. Dynamic background subtraction was turned off. Once an ion had been fragmented through MS/MS, its mass and isotopes were excluded from further MS/MS fragmentation for 15 s. The ions were fragmented in the collision cell using rolling collision energy, and CES was set to 5.

Three IDA MS raw files were combined and subjected to database searches in unison using ProteinPilot software v. 5.0 (AB SCIEX; Framingham, US) with the Paragon algorithm. The samples were input as unlabeled samples with the following parameters: iodoacetamide cysteine alkylation, digestion by trypsin and no special factors. The searches were conducted through identification efforts in a UniProt Swiss-Prot database (downloaded in September 2014, with 23906 protein sequence entries) containing whole *Gallus gallus* proteins. A false discovery rate analysis was also performed. The output of these searches, in the form of a group file, was used as the reference spectral library and contained the following information required for targeted data extraction: UniProt accessions, stripped peptide sequences, modified peptide sequences, Q1 and Q3 ion detection, retention times, relative intensities, precursor charges, fragment types, scores, confidence, and decoy results.

### SWATH-MS analysis and targeted data extraction

Eighteen samples (2 μg each) were subjected to the cyclic data independent acquisition (DIA) of mass spectra using a variable windows calculator (Supplemental Table 7) and the SWATH acquisition method editor (AB SCIEX; Framingham, US), similar to previously established methods^22^,^26^. For these experiments, the mass spectrometer was operated using a 0.05 s survey scan (MS). The subsequent MS/MS experiments were performed on all precursors in a cyclic manner using an accumulation time of 0.05 s per SWATH window for a total cycle time of 3.552 s. Ions were fragmented for each MS/MS experiment in the collision cell using rolling collision energy, and CES was set to 10.

The spectral alignment and targeted data extraction of DIA samples followed the Haverland *et al.* method^61^ and were performed using PeakView v.2.1 (AB SCIEX; Framingham, US) with the reference spectral library. All DIA files were loaded and exported in .txt format in unison using an extraction window of 20 min and the following parameters: six peptides/protein, six transitions/peptide, peptide confidence level of >99%, excluded shared peptides, and XIC width set at 50 ppm. This export procedure generated three distinct files containing the quantitative output for (1) the peak area under the intensity curve for individual ions, (2) the summed intensity of individual ions for a given peptide, and (3) the summed intensity of peptides for a given protein. For each protein, six individual ion intensities summed as peptide intensity, six peptides intensities summed as protein intensity. Mean of all biological and technical replicates was used to compare different group proteins.

### Using MRM and Western blotting to confirm protein changes discovered with SWATH technology

Twenty-six differentially expressed proteins that were previously analyzed via the SWATH method were selected for verification using the MRM method. In the MRM analysis, three technical and biological replicates were carried out for both control and heat treatment samples. The liquid chromatography instrument and settings employed were similar to the SWATH analysis, except for the stepwise gradient parameters (0–1 min, 95% A; 1–1.1 min, 90% A; 1.1–60 min, 80% A; 60–70 min, 76% A; 70–75 min, 50% A; 75–75.5 min, 20% A; 75.5–80.5 min, 20% A; 80.5–81 min, 95% A; 81–90 min, 95% A). A QTRAP® 6500 (AB SCIEX; Framingham, US) mass spectrometer with a nano-electrospray ionization source controlled by Analyst 1.6 software (AB SCIEX; Framingham, US was used for all scheduled LC–MRM/MS analyses. All acquisition and quantitation parameters were extracted and optimized from the group file of the reference spectral library using Skyline 2.6 software (Supplemental Table 8). The MRM acquisition methods were constructed employing five ion pairs per peptide, the declustering potential (DP), and collision energy (CE) voltages.

Western blotting was performed on 6 proteins from the three biological replicates using antibodies against the following proteins: HSPA5 (1:1,500, Thermo), CYP3A7 (1:2,000, Thermo), FADS1 (1:1,500, Thermo), NDUFA8 (1:5000, Thermo), GSTA2 (1:2,000, Thermo) and GAPDH (1:3,000, CST). The signal intensities of proteins were acquired using ChemiDoc XRS (Bio-Rad, USA) and the gray values of each protein band were calculated using Quantity One Software.

### Verifying the changes of proteins in ERK signal pathway and metabolites

Total RNA was extracted using RNAprep pure Tissue Kit (TIANGEN, DP431) following the manufacturer protocol. The concentration of RNA in each sample was determined by NanoDrop Spectrophotomer (ND-1000, Gene company Ltd) and the integrity of total RNA was checked by denatured RNA electrophoresis. First strand cDNA synthesis was completed using the qRT-PCR kit (TIANGEN, FP302) following the manufacturers protocol. Transcriptional expression of five genes of *MAPKs* superfamily (*MAPK1, MAPK6, MAPK9, MAPK11* and *MAPK14*) was quantified by real-time PCR. Primers (Tsingke, Beijing) used in the PCR reactions were designed using Primer 3 (http://bioinfo.ut.ee/primer3-0.4.0/). Detailed information on primer sequences is given in Supplemental Table 9. PCR reactions (20 μl) contained 1 μl of 5x diluted cDNA, 250 nM of each primer, and PCR buffer master mix from the Quant qRT-PCR kit (TIANGEN, FP302) were carried out using an ABI 7500 Real-Time PCR Detection System (Life Technologies). Each PCR reaction was conducted in triplicate. The geometric mean of internal references, β-actin and GAPDH, were used to normalize the expression of targets genes.

The fresh livers of broiler were quickly lyophilized at −50 °C. 0.1 g dry liver tissues were homogenized with 1 mL acetonitrile +0.1% formic acid, adding 50 ng/ml norleucine as the internal standard. After a thorough homogenization, the homogenates of liver tissue were centrifuged at 12,000 *g* for 10 min at 4 °C. Supernatants were carefully transferred to 2 mL tubes and then injected onto the LC-MS/MS system (Waters Xevo TQ) by an autosampler for subsequent analysis. In the LC-MS/MS analysis, Methionine was separated using ACQUITY UPLC BEH C18 1.7 μm (2.1 × 50 mm). Creatine, Dopamine and 3-Methoxytyramine were separated using ACQUITY UPLC BEH Amide 1.7 μm (2.1 × 100 mm).

### Statistical and GO analysis

The protein quantitative data were organized using Microsoft Excel 2010. The analysis of coefficients of variation for replicates and *t*-tests between the control and heat treatment groups were performed using Microsoft Excel 2010. The correlation and linear regression of repeats were analyzed with GraphPad software version 6.01. GO analysis was performed using Blast2GO® software^62^. Pathway analysis was performed using Ingenuity Pathway Analysis (IPA).

## Additional Information

**How to cite this article**: Tang, X. *et al.* Label-free Quantitative Analysis of Changes in Broiler Liver Proteins under Heat Stress using SWATH-MS Technology. *Sci. Rep.* **5**, 15119; doi: 10.1038/srep15119 (2015).

## Supplementary Material

Supporting figures

Supporting tables

## Figures and Tables

**Table 1:** The statistical analysis of the quantitated result of broiler liver proteins via SWATH technology.

Sample groups	Control			Heat stress		
	C1	C2	C3	H1	H2	H3
The protein number in DDA protein library	4271					
The peptide number in DDA protein library	36073					
The number of identified proteins in SWATH test	3646					
The number and ratio of quantitative protein, CV ≤ 25% among technologic repeats	3445(94.5%)	3104(85.1%)	3372(94.5%)	3389(93.0%)	3316(91.0%)	3360(94.5%)
The number of quantitative proteins, which had been quantitated in all eighteen repeats.	2481					
The number and ratio of quantitative protein, CV ≤ 25% among biologic repeats	2138(86.2%)			2003(80.7%)		
The final number of quantitative protein	2377					

**Table 2:** Summary of differential proteins discussed in the Discussion section of this manuscript.

Entry ID	Protein names	Protein description	Location	Type(s)	Fold Change
ERK interactors					
E1C619	ABCA1	ATP-binding cassette, sub-family A (ABC1)	Plasma Membrane	transporter	−1.342
E1BSH9	BHMT	betaine—homocysteine S-methyltransferase	Cytoplasm	enzyme	+2.022
O57389	CNP	2’,3’-cyclic nucleotide 3’ phosphodiesterase	Cytoplasm	enzyme	−1.446
H9L027	CTSG	cathepsin G	Cytoplasm	peptidase	−1.549
A7E3K8	CYBB	cytochrome b-245, beta polypeptide	Cytoplasm	enzyme	−1.500
Q5F494	DGKZ	diacylglycerol kinase, zeta	Cytoplasm	kinase	−1.406
E1C688	HPGD	hydroxyprostaglandin dehydrogenase 15	Cytoplasm	enzyme	+1.304
F1NWB7	HSP90B1	heat shock protein 90kDa beta	Cytoplasm	other	+1.416
F1NGX1	ITGAV	integrin, alpha V	Plasma Membrane	ion channel	−1.377
E1C6C0	MEMO1	mediator of cell motility 1	Cytoplasm	other	−1.382
F1P0K7	PFKFB4	6-phosphofructo-2-kinase/fructose-2,6-biphosphatase 4	Cytoplasm	kinase	−1.335
E1C6E1	PLCG2	phospholipase C	Cytoplasm	enzyme	−1.413
F1N8W3	POSTN	periostin, osteoblast specific factor	Extracellular Space	other	+1.686
F1NW88	STAB2	stabilin 2	Plasma Membrane	transmembrane	−1.370
Lipid metabolism					
E1C619	ABCA1	ATP-binding cassette, sub-family A (ABC1)	Plasma Membrane	transporter	−1.342
E1BU39	ACOT1	acyl-CoA thioesterase 1	Cytoplasm	enzyme	−1.442
E1BVK8	ACOT8	acyl-CoA thioesterase 8	Cytoplasm	enzyme	−1.514
Q5ZM10	ACOX1	acyl-CoA oxidase 1, palmitoyl	Cytoplasm	enzyme	−1.332
F1NXK4	ACSF3	acyl-CoA synthetase family member 3	Cytoplasm	enzyme	−1.905
Q6B842	CPT1A	carnitine palmitoyltransferase 1A	Cytoplasm	enzyme	−1.359
F1NSE3	CRAT	carnitine O-acetyltransferase	Cytoplasm	enzyme	−1.374
Q05423	FABP7	fatty acid binding protein 7, brain	Cytoplasm	transporter	−1.555
E7EDS8	FADS1	fatty acid desaturase 1	Plasma Membrane	enzyme	−1.836
H9L1A8	GLTP	glycolipid transfer protein	Cytoplasm	transporter	−1.662
F1P1E7	HMGCL	3-hydroxymethyl-3-methylglutaryl-CoA lyase	Cytoplasm	enzyme	−1.471
F1NGX1	ITGAV	integrin, alpha V	Plasma Membrane	ion channel	−1.377
Q08156	KIT	v-kit Hardy-Zuckerman 4 feline sarcoma	Plasma Membrane	transmembrane receptor	−1.331
Q5ZMB9	LYN	LYN proto-oncogene, Src family tyrosine kinase	Cytoplasm	kinase	−1.322
F1NIX4	MECR	Mitochondrial trans-2-enoyl-CoA reductase	Cytoplasm	enzyme	−1.316
E1BQN0	NDUFS6	NADH dehydrogenase Fe-S protein 6,	Cytoplasm	enzyme	−1.499
E1BSH2	SLCO1A2	solute carrier organic anion transporter family	Plasma Membrane	transporter	+1.385
E1BQB7	SMPD4	sphingomyelin phosphodiesterase 4	Cytoplasm	enzyme	−1.316
R4GH73	THEM4	thioesterase superfamily member 4	Plasma Membrane	enzyme	−1.615
Amino acid metabolism					
E1BSH9	BHMT	betaine—homocysteine S-methyltransferase	Cytoplasm	enzyme	+2.022
E1BTA0	COMT	catechol-O-methyltransferase	Cytoplasm	enzyme	−1.516
E1BV90	DDC	dopa decarboxylase	Cytoplasm	enzyme	+1.458
R4GIK2	GAMT	guanidinoacetate N-methyltransferase	Cytoplasm	enzyme	−1.316
Q6PKI8	PAH	phenylalanine hydroxylase	Cytoplasm	enzyme	+1.467
Cell inflammatory and immune responses					
E1C619	ABCA1	ATP-binding cassette, sub-family A (ABC1)	Plasma Membrane	transporter	−1.342
P11501	HSP90AA1	heat shock protein 90 kDa alpha	Cytoplasm	enzyme	+1.520
P51890	LUM	lumican	Extracellular Space	other	+1.347
Q5ZMB9	LYN	LYN proto-oncogene, Src family tyrosine kinase	Cytoplasm	kinase	−1.322
Q2PUH1	PRKAA1	protein kinase	Cytoplasm	kinase	−1.320

Notes: + means upregulated; − means downregulated.
