# Supplementary material for: Label-free Quantitative Analysis of Changes in Broiler Liver Proteins under Heat Stress using SWATH-MS Technology
Source: Sci Rep. 2015 Oct 13;5:15119. doi: 10.1038/srep15119 (PMC4602270; doi:10.1038/srep15119)
Supplement: Supporting figures [file srep15119-s1.doc]

**Title:** Label-free Quantitative Analysis of Changes in Broiler Liver Proteins under Heat Stress using SWATH-MS Technology

**Authors**: Xiangfang Tang, Qingshi Meng, Jie Gao, Sheng Zhang, Hongfu Zhang, Minhong Zhang

**Supporting Information**

**Supporting figures information**

**Supplemental Figure 1**


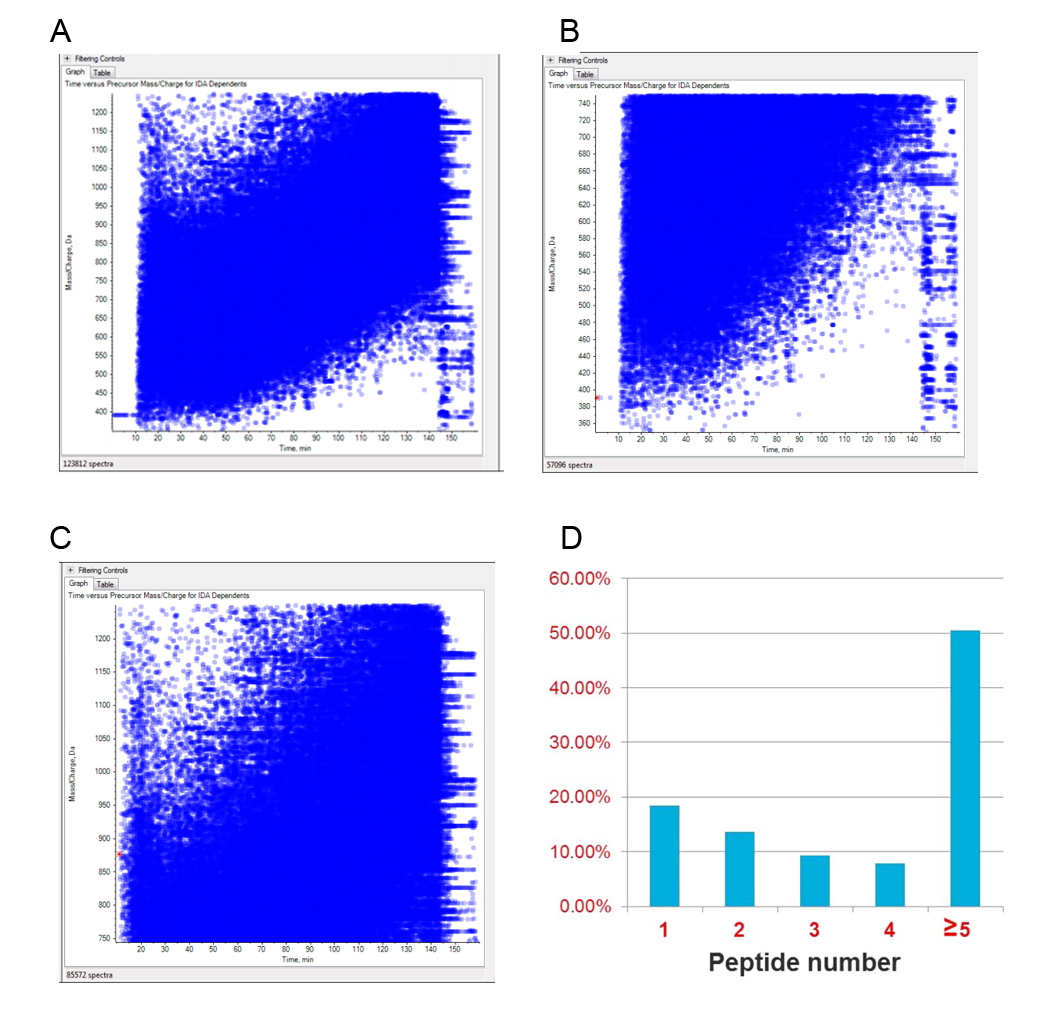


**The number of MS/MS spectra in different runs and identified peptide analysis**

1. A total of 123,812 high-quality MS/MS spectra was collected in Run1 (*m/z* 350-1,250); B. A total of 57,096 high-quality MS/MS spectra was collected in Run2 (*m/z* 350-750); C. A total of 85,572 high-quality MS/MS spectra was collected in Run3 (*m/z* 745-1,250); D. A total of 4,271 proteins were identified at 1% FDR, and approximately 50% of the proteins matched more than five peptides.

**Supplemental Figure 2**


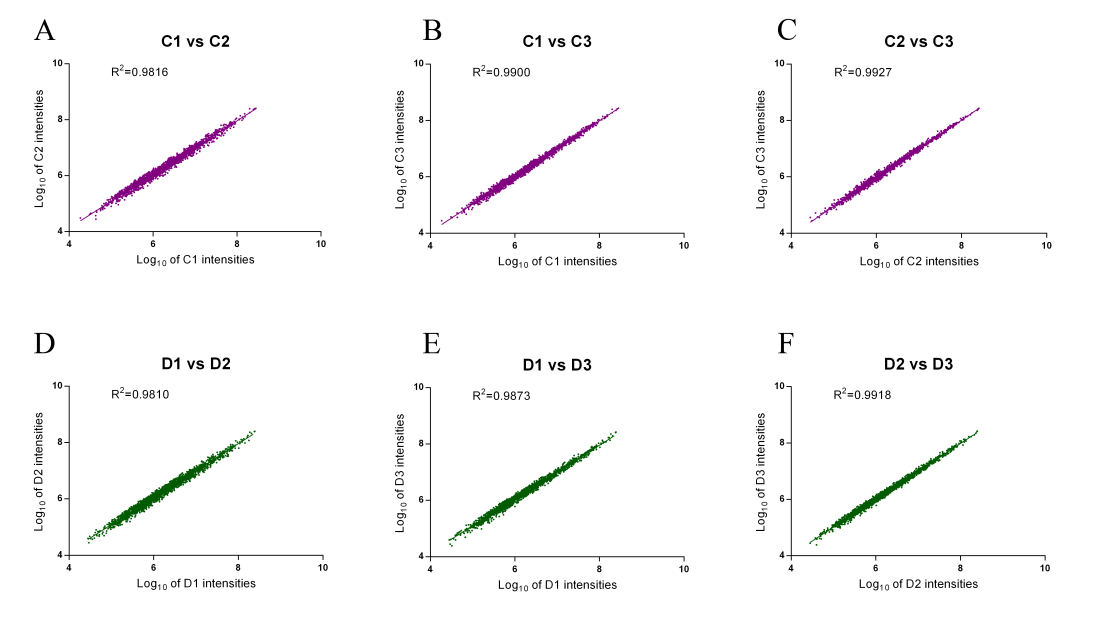


**The analysis of variance to determine quantitative reproducibility between biological repeats**

A-C shows the variance analysis of three biological repeats in control group, and the R^2^ values for two biological repeats were 0.9816, 0.9900 and 0.9927, respectively; D-F shows the variance analysis of three biological repeats in heat treatment group, and the R^2^ values for two biological repeats were 0.9810, 0.9873 and 0.9918, respectively. The analysis result suggesting the quantitative SWATH data from replicates were quite reproducible.

**Supplemental Figure 3**

**
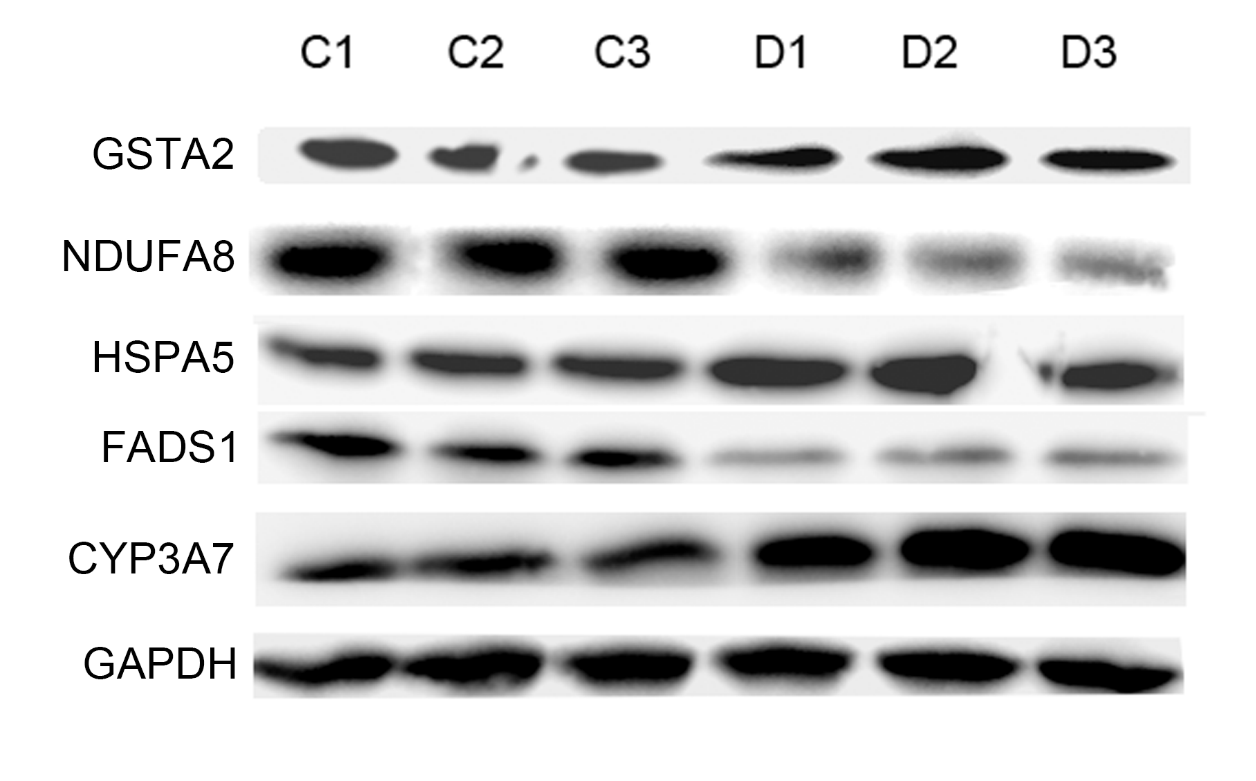
**

**The results of the Western blot analysis the abundance of five proteins.**

The abundance of HSPA5, CYP3A7, FADS1, NDUFA8 and GSTA2 proteins were analyzed by classical method of Western blotting. The GAPDH protein was as a internal reference.
